# Succinate-mediated reactive oxygen species production in the anoxia-tolerant epaulette (*Hemiscyllium ocellatum*) and grey carpet (*Chiloscyllium punctatum*) sharks

**DOI:** 10.1098/rsbl.2023.0344

**Published:** 2023-10-11

**Authors:** Jules B. L. Devaux, Anthony J. R. Hickey, Gillian M. C. Renshaw

**Affiliations:** ^1^ School of Biological Sciences, The University of Auckland, Private Bag 92019, Auckland Mail Centre, Auckland 1142, New Zealand; ^2^ Hypoxia and Ischemia Research Unit School of Allied Health Sciences, Griffith University, Gold Coast campus, Queensland 4222, Australia

**Keywords:** reactive oxygen species, anoxia, mitochondria, respirometry

## Abstract

Anoxia/re-oxygenation (AR) results in elevated unchecked oxidative stress and mediates irreversible damage within the brain for most vertebrates. Succinate accumulation within mitochondria of the ischaemic brain appears to increase the production of reactive oxygen species (ROS) upon re-oxygenation. Two closely related elasmobranchs, the epaulette shark (*Hemiscyllium ocellatum*) and the grey carpet shark (*Chiloscyllium punctatum*) repeatedly experience near anoxia and re-oxygenation in their habitats and have adapted to survive AR at tropical temperatures without significant brain injuries. However, these anoxia-tolerant species display contrasting strategies to survive AR, with only *H. ocellatum* having the capacity to supress metabolism and *H. ocellatum* mitochondria the capacity to depress succinate oxidation post-AR. We measured oxygen consumption alongside ROS production mediated by elevated succinate in mitochondria of permeabilized cerebellum from both shark species. Although mitochondrial respiration remained similar for both species, the ROS production in *H. ocellatum* was half that of *C. punctatum* in phosphorylating and non-phosphorylating mitochondria. Maximum ROS production in *H. ocellatum* was mediated by succinate loads 10-fold higher than in *C. punctatum* mitochondria. The contrasting survival strategies of anoxia-tolerant sharks reveal the significance of mitigating ROS production under elevated succinate load during AR, shedding light on potential mechanisms to mitigate brain injury.

## 1. Introduction

**Figure 1.**
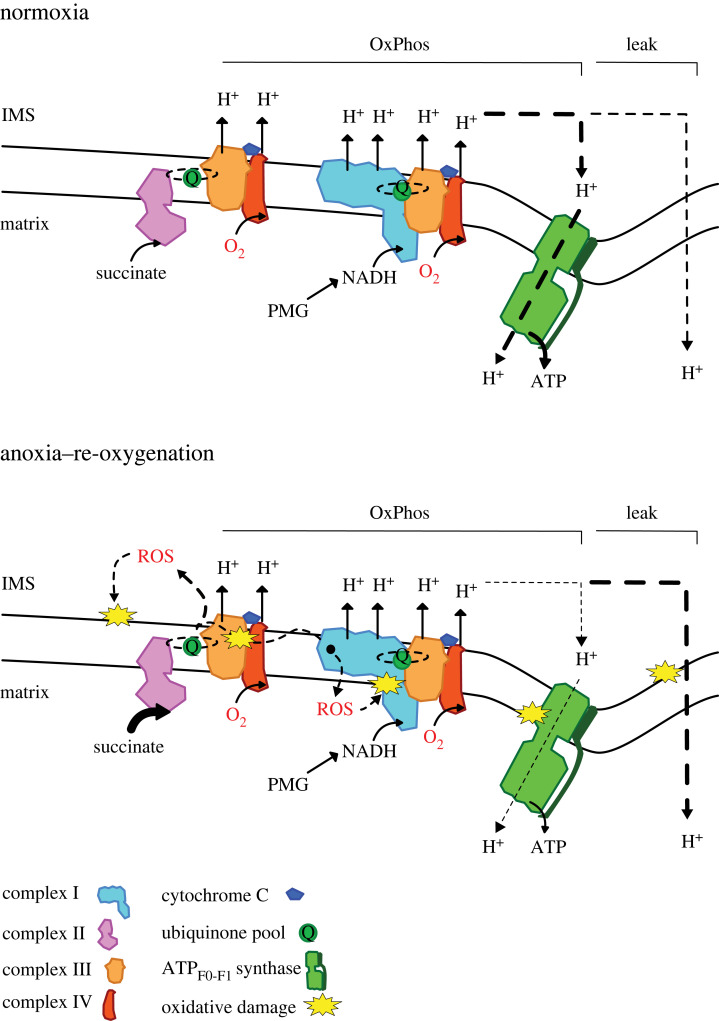
Representation of the mitochondrial electron transport system under normoxia, and post anoxia–re-oxygenation. Electron input to the electron transport system may occur at complex I from NADH-linked substrates such as pyruvate, malate and glutamate (PMG), and at complex II from the metabolite succinate. Electrons flow to oxygen via complexes of the transport system as this mediates proton pumping to the inter-membrane space (IMS). Protons are utilized by the ATP_F0-F1_ synthase via the process known as oxidative phosphorylation (OxPhos). A portion of protons may not be utilized by the ATP_F0-F1_ and ‘Leak’ directly to the mitochondrial matrix. However, in anoxia, succinate accumulates in mitochondria and can feed excess electrons into the transport system during re-oxygenation, which exacerbate reactive oxygen species (ROS) production. The latter can damage mitochondrial components and increase Leak, at the detriment of OxPhos. Raising the question: have anoxia-tolerant species evolved mechanisms to overcome these events enabling them to survive cyclic environmental anoxia–re-oxygenation? Figure adapted from (Devaux *et al.* [12]).

Hypoxia and anoxia-tolerant species have evolved a multitude of strategies to survive anoxia in their natural environment [1,2]. While episodes of hypoxia or anoxia are well tolerated by these species, reoxygenation presents another challenge because it incurs serious injuries and may cause irreversible damage in non-tolerant species [3]. Succinate is a metabolic intermediate that appears to play a key role in the cellular response to ischaemia–reperfusion injuries in mammals [4–11]. During oxygen deprivation, succinate accumulates due to impaired function of mitochondrial complexes. Upon reperfusion, succinate is rapidly oxidized, leading to a reversal of electron flow associated with a burst of reactive oxygen species (ROS) production, capable of causing cellular damage [11], as illustrated in figure 1.

To evade excess oxidative damage, a few hypoxia and anoxia-tolerant species supress ROS production [13,14], and this can be associated with an increase in antioxidant defences [15]. The epaulette shark (*Hemiscyllium ocellatum*) and the grey carpet shark (*Chiloscyllium punctatum*) are two closely related elasmobranches of the carpet shark family that can tolerate prolonged anoxia at tropical temperatures [16]. In response to anoxia, *C. punctatum* rapidly increased its haematocrit, likely representing splenic contraction to supply metabolically active organs with O_2_ from stored red blood cells [17]. In contrast, the more anoxia-tolerant *H. ocellatum* is capable of metabolic depression and neuronal hypometabolism [18,19] and does not increase its haematocrit when under stressful conditions [12]. Additionally, *H. ocellatum* responds to hypoxia pre-conditioning, after which it more readily enters ventilatory and metabolic depression [20]. Recently, it has been demonstrated that *ex vivo* mitochondrial plasticity occurs in the cerebellum from *H. ocellatum* evidenced by the suppression of succinate dehydrogenase (i.e. mitochondrial complex II 'CII') activity and readjustment of electron transfer pathways in response to exposure to anoxia/re-oxygenation (AR) [21]. While this strategy may prevent reverse electron flow in response to highly oxidized succinate, the ROS production in the brain mitochondria of this robust species has yet to be compared with a similar anoxia-tolerant species such as *C. punctatum*.

Based on the contrasting physiological responses of *H. ocellatum* and *C. punctatum* to AR, we sought to assess the mitochondrial response to elevated levels of succinate and the resultant level of ROS production by cerebella mitochondria of both shark species. Using respirometry coupled with fluorimetry, we tested the hypothesis that mitochondria from *H. ocellatum* would better manage succinate-derived ROS relative to *C. punctatum* mitochondria, and that the resulting electron leakage resulting from succinate oxidation would be avoided in *H. ocellatum* cerebellum. This study aims to address the contrasting strategies displayed by two anoxia-tolerant sharks at the mitochondrial level and is the first to provide insights into mitochondria ROS production in *C. punctatum*.

## 2. Material and methods

Ten adult grey carpet sharks (*Chiloscyllium punctatum*) and 10 adult epaulette sharks (*Hemiscyllium ocellatum*) were supplied by Sea World (Main Beach, Gold Coast, Australia) or by Cairns Marine (Cairns, Australia), respectively. Sharks were held under standard condition of temperature, photoperiod and feeding as described for similar individuals in [21]. After two weeks acclimation, shark euthanasia and cerebellum extraction and permeabilization was undertaken as described previously [21]. Briefly, sharks were euthanized by the addition of freshly prepared benzocaine and the cerebellum was immediately immersed in ice-cold relaxing buffer. Cellular permeabilization was undertaken on approximately 10–20 mg pieces of cerebellum for the respirometry and fluorimetry assays.

A substrate-uncoupler-inhibitor titration (SUIT) protocol (table 1) was employed to reflect (i) succinate build-up in non-phosphorylating mitochondria (i.e. anoxic mitochondria) and the (ii) the ROS production mediated by the high oxidation rate of succinate upon re-oxygenation, as observed in other vertebrates [11]. Permeabilized cerebellum was added to the 2 ml chamber of a high-resolution respirometer OROBOROS O2ks containing aerated respiration medium thermostatically controlled at 20°C (approx. 262 µM O_2_ at 101.5 kPa). The O_2_ decline over time due to mitochondria respiration (*JO*_2_) was measured in real time and was recorded using DatLab 7 analysis software. The ROS production was measured simultaneously with fluorimeters, as previously described [14], and corrected with sample-free background shift in fluorescence.

**Table 1.** Representative trace of the substrate-inhibitor-uncoupler protocol for simultaneous measurement of mitochondrial respiration and ROS production. The effect of accumulating succinate on the mitochondrial ROS production was assessed in permeabilized cerebellum of grey carpet sharks and epaulette sharks. ROS production was calibrated within the chamber of OROBOROS oxygraphs by the titration of H_2_O_2_ prior the addition of the sample. After around 20 min of recovery time, succinate was titrated up to 10 mM to put mitochondria into Leak state (Leak_S_) and to stimulate maximum ROS production mediated by CII. The addition of saturated ADP stimulates oxidative phosphorylation mediated by succinate (OxPhos_S_), which maximum capacity (OxPhos_PMGS_) was reached by the subsequent addition of NADH-linked substrates (pyruvate, malate and glutamate). Respiration not attributed to OxPhos (i.e. Leak_PMGS_) was then measured with the addition of the ATP_F0-F1_ inhibitor oligomycin. Finally, mitochondria were uncoupled from respiration with the titration of CCCP to measure the maximum capacity of the mitochondrial electron transport system (ETS_PMGS_).

step	compound	concentration	coupling state	ET pathway state	function
1	HRP	1 U ml^−1^			H_2_O_2_
	+ SOD	5 U ml^−1^			fluorescence
	+ Amplex	10 µM			calibration
	UltraRed™	0.1 µM (×3)			
	+ H_2_O_2_ (x3)				
2	sample	∼ 10–20 mg	ROX		
3	succinate titration		Leak_S_	S	substrate of complex II
4	ADP	5 mM	OxPhos_S_	S	substrate of ATP synthase
5	pyruvate	5 mM	OxPhos_PMGS_	NADH + S	NADH-linked substrates
	+ malate	0.1 mM			
	+ glutamate	10 mM			
6	oligomycin	5 µM	Leak_PMGS_	NADH + S	ATP synthase inhibitor
7	CCCP	0.5 µM per step	ETS_PMGS_	NADH + S	uncoupling of mitochondrial respiration

**Figure 2.**
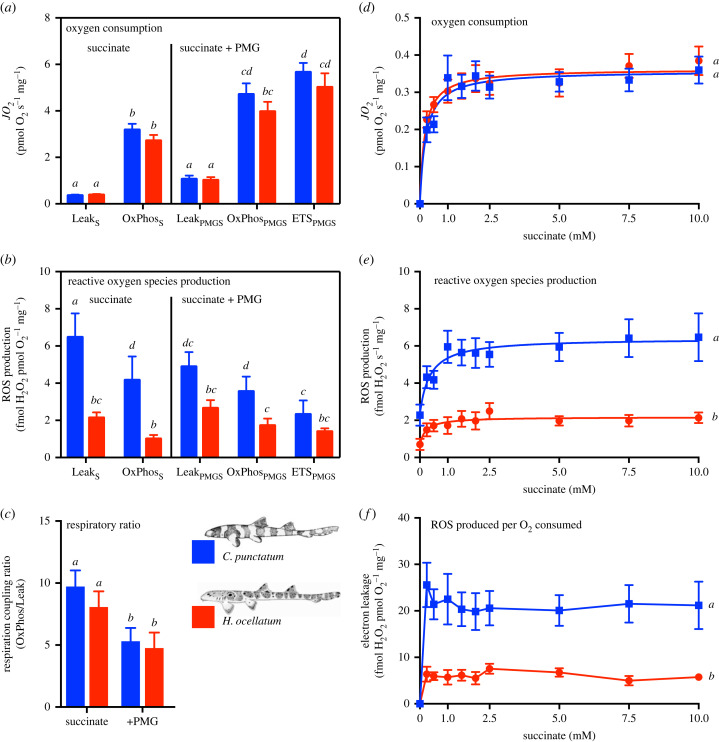
Mitochondrial respiration and ROS production in response to mitochondrial substrate-dependence in permeabilized cerebellum of *C. punctatum* and *H. ocellatum*. While the mitochondrial respiration (*JO*_2_) was similar between species (*a*), the ROS production was higher in *C. punctatum* when mediated by succinate (*b*). (*c*) Mitochondria in both sharks are approximately 1.6× more coupled to OxPhos in the presence of succinate only relative to additional pyruvate, malate and glutamate (+PMG). Refer to the table 1 legend for the description of the *x*-axis. In the absence of other substrates and ADP, succinate was titrated in chambers of oxygraphs containing permeabilized cerebellum. These conditions aimed to reflect those experienced by anoxic brain *in vivo*. Simultaneously with mitochondria respiration (*d*), the ROS production was measured and expressed in absolute values (*e*) or relative to *JO*_2_ as a measure of electron leakage from the ETS (*f*). Fitted curves in (*d*) and (*e*) were extracted using the least-squares method based on a dose-stimulation model. From 0.25 mM succinate, *C. punctatum* mitochondria produce more ROS (absolute or relative to respiration) than *H. ocellatum* mitochondria. Results presented as mean of *n* = 10 ± s.e.m. Two-way ANOVA (*a,b,c*) and two-way ANOVA repeated measures (*d,e,f*) followed by Tukey's *post-hoc* tests were performed to test for differences at *p* < 0.05 between sharks and mitochondrial states and shown by uncommon letters.

Two-way ANOVA tests were performed to test for species or substrate/inhibitor effects on respiration and on ROS production rates (figure 2*a–c*). For succinate titration (figure 2*d*–*f*), fitted curves for respiration and ROS were extracted using the least-squares method based on a dose-stimulation model. Two-way ANOVA with repeated measures tests were then performed to test for main effects by species or succinate concentration. Post-hoc tests with multiple comparison (Tukey correction) were used to assess significant differences chosen as *p* < 0.05: between species; mitochondria states; and between succinate concentrations. Raw data and statistical analysis are publicly available at the University of Auckland Figshare repository: (doi:10.17608/k6.auckland.22144289) [22].

## 3. Results

No difference in mitochondria O_2_ flux was apparent between *C. punctatum* and *H. ocellatum* permeabilized cerebellum (figure 2*a*; *F*_1,9_ = 1.075, *p* = 0.33). When supported by succinate, OxPhos rates supported around 70% of OxPhos supported by additional NADH-linked substrates in both species (*p* < 0.0001). Respiratory coupling ratio supported by succinate (RCR) were twice greater than when supported with additional NADH-linked substrates (*F*_1,9_ = 31.2, *p* < 0.001; figure 2*c*), with no difference between species (*p* = 0.48). The exogeneous titration of succinate mediated a similar increase in respiration in cerebellar mitochondria of both shark species (*p* = 0.76; figure 2*d*).

ROS production was two- to threefold higher in cerebellar mitochondria from *C. punctatum* than those from *H. ocellatum* (*F*_1,9_ = 8.95, *p* = 0.02; figure 2*b*) with an interaction between species and mitochondria state (*F*_4,36_ = 3.46, *p* = 0.02). In *H. ocellatum*, ROS production was similar across all mitochondrial states, (*p* > 0.38), with less ROS production in Leak_S_ relative to Leak_PMGS_, suggesting that complex I most likely mediated the greatest portion of ROS when mitochondria were not phosphorylating. In contrast, the highest ROS production was observed when *C. punctatum* mitochondria were in Leak_S_ fuelled by succinate (*p* < 0.06).

In non-phosphorylating mitochondria, increments of exogenous succinate increased the ROS production from 0.25 mM in both species (main effect *F*_1,162_ = 7.21, *p* < 0.0001; figure 2*e*). The ROS production was however threefold higher in *C. punctatum* mitochondria relative to *H. ocellatum* mitochondria (*F*_1,18_ = 27.9, *p* < 0.0001). In *C. punctatum* mitochondria, 1 mM succinate was sufficient to mediate the highest electron leakage (*p* < 0.0001), while in *H. ocellatum* mitochondria the maximum levels only achieved with a 2.5 higher level of succinate (i.e. 2.5 mM) (figure 2*f*; Tukey *post-hoc* test). While the absolute ROS production may reflect an overall potential for oxidative damage or stress, the normalization of ROS per O_2_ consumed also represents electron leakage and therefore efficiency of electron transport (figure 2*f*). *Chiloscyllium punctatum* mitochondria had a threefold higher electron leakage mediated by succinate oxidation relative to those of *H. ocellatum* (species main effect of *F*_1.18_ = 20.76, *p* = 0.0002). However, the portion of electron leakage remained constant in both shark species regardless of the succinate concentration (*F*_8.144_ = 0.41, *p* = 0.91; figure 2*f*).

## 4. Discussion

### (a) Oxygen flow in permeabilized cerebella

Mitochondria from permeabilized cerebella had similar respiration rates in both shark species. OxPhos rates in permabilized cerebella were however three-times greater than those previously measured in homogenized cerebella of these species [21]. In addition, *H. ocellatum* cerebella, the ETS flux was lower in permeabilized tissue relative to homogenates. Both homogenization and permeabilization preparations facilitate the exchange of soluble molecules between the medium and the cytosolic phase, including succinate to which most plasma membranes are impermeable [23]. However, permeabilization was chosen over homogenization for this study as this process washes the cytosol content and limits from the effect of endogenous substrates, including succinate, which could affect flux analysis [24]. Despite the difference in absolute rates, coupling efficiencies were similar and greater than 5, indicating preservation of the mitochondria integrity in both preparations. Both sharks demonstrated a 20% reserve capacity suggesting that they have the ability to sustain some damage on ETS without impacting OxPhos rates (i.e. ATP production). This is likely advantageous during and after episodes of oxygen limitation, which are known to cause damage to the ETS in most anoxia-sensitive vertebrates [14,25].

The additive effect of convergent electron transfer from NADH-linked and CII should result in a competition for the Q-pool. In SUIT protocols, the sequential addition of NADH-linked and CII substrates is thought to monopolize a portion of the Q-pool, which then becomes less available to further substrates [24]. In this context, the maximum contribution of different substrates (NADH-linked or CII-linked) to OxPhos may be under-estimated when the Q-pool is partially reduced in the presence of pre-loaded mitochondrial substrates. In a previous study [21], succinate (10 mM) contributed to around 20 and 30% of *JO_2_* in the OxPhos state when in the presence of saturating NADH-linked substrates in *H. ocellatum* and *C. punctatum* mitochondria, respectively. Here, equally concentrated succinate could sustain 70% of OxPhos when no other additional substrates were present (figure 2*a*). The data also reveal that near-full CII capacity is used when the Q-pool does not compete with NADH-linked inputs, when compared to maximum CII flux [21]. In addition, coupling ratios indicate a greater O_2_ utilization when OxPhos is fuelled by succinate, relative to when NADH-linked substrates are also present. Notably, CII mediated respiration transfers of 40% less protons per O_2_ than NADH-linked-fuelled respiration. Therefore, to achieve similar ATP synthesis rates, more O_2_ is required when more CII is used to drive OxPhos. In cerebellar mitochondria from *C. punctatum*, succinate may therefore serve as a fuel reserve that can be used for OxPhos when NADH-linked substrates may become limiting. In contrast cerebellar mitochondria from *H. ocellatum* turn down OxPhos in the presence of excess succinate.

### (b) ROS production mediated by succinate

In mitochondria, ROS production is in part mediated by substrate levels, which alter redox state and mitochondrial phosphorylation state [26]. All of which are compromised by AR and this changes the ROS output that may serve as signalling factors to mediate redox regulation and oxidative damage repair [27,28]. In *C. punctatum* mitochondria, the ROS production was the highest in non-phosphorylating mitochondria with succinate (Leak_S_). In contrast, the ROS production from *H. ocellatum* mitochondria appeared higher when in Leak_S_ with succinate and NADH-linked inputs (figure 2*b*). Although high leak flux has been shown to decrease the ROS production [29], high ROS production is typical of a State-4 like condition [26,30]. Overall, the ROS production was lower in *H. ocellatum* mitochondria. With similar O_2_ flux, this suggests that electrons appear to be more efficiently channelled in the ETS to O_2_ in *H. ocellatum* cerebellar mitochondria. We also note that additional NADH-linked substrate mediate higher proton Leak, as suggested by greater RCR when OxPhos is fuelled by succinate only (figure 2*c*).

In the brain, succinate has been measured in the range of 0–0.5 mM in normoxia and this increases to several mM during anoxia in vertebrates [31,32], including the anoxia-tolerant crucian carp [33]. Therefore, we titrated succinate into the chambers to match the range of concentrations encountered in each of these physiological conditions. At all succinate levels, the ROS production was higher in *C. punctatum* mitochondria*,* the maximal production was reached at 0.1 mM of succinate (figure 2*d*). Succinate concentrations encountered in normoxic brain could be expected to be lower than 0.5 mM [34], the ROS production was approximately 4 fmol H_2_O_2_ s^−1^ mg^−1^, in phosphorylating mitochondria (cf. OxPhos rates, figure 2*b*). In addition, approximately 2.2% of the electrons supplied from succinate oxidation were directed to ROS production, which likely represents a small yet measurable loss of coupling of the energy from electron transfer to proton pumping, previously reported [35]. In addition, the NADH-linked mediated *JO_2_* in *C. punctatum* mitochondria was altered after AR, as we reported previously [21]. In accordance with observations on mammalian brain [11], a reverse electron flow would be expected from the succinate induced electron leakage, in turn this would decrease the capacity of NADH-linked to sustain respiration in cerebellar mitochondria from *C. punctatum,* as observed in [21].

Conversely, the ROS production from *H. ocellatum* cerebella reached a plateau at much higher (1 mM) succinate concentration, and electron leakage remained constant and low at 0.8% of the total electron flow fuelled by succinate oxidation, thus regardless of succinate concentrations, the mitochondria from *H. ocellatum* showed less evidence of reverse electron flow or increased ROS production. This indicates that in the eventuality of oxygen-limited succinate accumulation, oxidative damage during re-oxygenation would be averted regardless of succinate levels exceeding 1 mM. This is in accord with the decrease in succinate oxidation rate (CII mediated respiration) observed after AR *ex vivo* [21].

In addition to enhanced active proton Leak rates post AR [21], mitochondria from the cerebellum of *H. ocellatum* benefit from two mechanisms that avert oxidative damage mediated by elevated succinate (i) partial inhibition of CII respiration post AR [21], (ii) low and steady ROS production along with low electron leakage even at high succinate levels. Interestingly, the AR protective responses measured in these two ancient lineages of sharks have been observed in a number of other lineages: (i) selected hypoxia-tolerant *Drosophila,* which displayed an enhanced Leak, a lower CII activity and a lower mitochondria ROS production [36]; (ii) chemical inhibition of CII, in murine heart, was shown to be a beneficial protectant against ischaemia–reperfusion via decrease in ROS production [10]; (iii) succinate regulation has been reported in the hypoxia-tolerant ground squirrel [37]; and (iv) a decrease is ROS production post AR is a common adaptation among ectotherms species such as the trout [38] and the killifish [15].

## 5. Conclusion

This study is the first to investigate the production of ROS in the cerebellar mitochondria of *H. ocellatum* and *C. punctatum* during anoxia and re-oxygenation, and how graded excess succinate affects ROS production *ex vivo*. Mitochondrial responses may not strictly corelate with mitochondrial response *in situ*, where regulations at higher biological levels could occur. Nevertheless, our findings indicate that *C. punctatum* had higher levels of ROS production, likely due to higher electron leakage caused by high succinate oxidation rates post AR *in vitro*. By contrast, *H. ocellatum* had lower ROS production due to decreased succinate oxidation rates post AR, which enabled conservation of respiration and oxidative phosphorylation capacities. While mitochondrial plasticity in the cerebella of both sharks was assessed in *ex vivo* settings, the observed mitochondrial response aligns with the contrasting ecophysiological responses of the two species to anoxia and re-oxygenation that are presented in shallow coastal environments. These two distinct strategies used to achieve anoxia-tolerance at the subcellular level among elasmobranchs may assist with the design of potential interventions to mitigate brain damage in other species and even humans via controlling CII activity and unchecked ROS production.

## Data Availability

Data supporting this manuscript are available at the University of Auckland Repository: https://doi.org/10.17608/k6.auckland.22144289 [22].
